# Mycorrhizal Symbiosis Triggers Local Resistance in Citrus Plants Against Spider Mites

**DOI:** 10.3389/fpls.2022.867778

**Published:** 2022-07-01

**Authors:** María Manresa-Grao, Julia Pastor-Fernández, Paloma Sanchez-Bel, Josep A. Jaques, Victoria Pastor, Víctor Flors

**Affiliations:** ^1^Metabolic Integration and Cell Signaling Laboratory, Plant Physiology Section, Unidad Asociada al Consejo Superior de Investigaciones Científicas, Department of Biology, Biochemistry and Natural Sciences, Universitat Jaume I, Castelló de la Plana, Spain; ^2^Department of Biology, Biochemistry and Natural Sciences, Universitat Jaume I, Castelló de la Plana, Spain

**Keywords:** arbuscular mycorrhiza, *Tetranychus urticae*, *Citrus aurantium*, mycorrhiza induced resistance, jasmonic acid, arbuscular mycorrhizas

## Abstract

Citrus plants are a highly mycotrophic species with high levels of fungal colonization. *Citrus aurantium* rootstocks typically show abundant root colonization by *Rhizophagus irregularis* three weeks after inoculation. Mycorrhizal symbiosis protects plants against multiple biotic stressors, however, such protection against spider mites remains controversial. We examined mycorrhiza-induced resistance (MIR) in citrus against the two-spotted spider mite *Tetranychus urticae*. Mycorrhized *C. aurantium* displayed reduced levels of damage in leaves and lower mite oviposition rates, compared to non-mycorrhized controls. Mycorrhization did not affect host choice of mites in Y-tube assays; of note, *C. aurantium* has innate strong antixenotic resistance against this mite. Analysis of metabolism pathways in mycorrhized citrus plants showed upregulated expression of the oxylipin-related genes *LOX-2* and *PR-3* early after infestation. Accordingly, jasmonic acid (JA), 12-oxo phytodienoic acid (OPDA), and JA-Ile concentrations were increased by mycorrhization. Non-targeted metabolomic analysis revealed the amino acid, oxocarboxylic acid, and phenylpropanoid metabolism as the three major pathways with more hits at 24 h post infection (hpi) in mycorrhized plants. Interestingly, there was a transition to a priming profile of these pathways at 48 hpi following infestation. Three flavonoids (i.e., malic acid, coumaric acid, and diconiferyl alcohol) were among the priming compounds. A mixture containing all these compounds provided efficient protection against the mite. Unexpectedly, systemic resistance did not improve after 72 h of primary infestation, probably due to the innate strong systemic resistance of *C. aurantium*. This is the first study to show that MIR is functional against *T. urticae* in locally infested citrus leaves, which is mediated by a complex pool of secondary metabolites and is likely coordinated by priming of JA-dependent responses.

## Introduction

The polyphagous spider mite *Tetranychus urticae* (Acari: Tetranychidae) has adapted to a wide host range through detoxification of plant defense compounds (Agut et al., 2018). Among the mites’ strategies to avoid plant toxins, the *T. urticae* genome contains multiple copies of genes encoding Cys-proteases that counteract plant proteinase inhibitors (Santamaría et al., 2015). In addition, two-spotted spider mites adapt rapidly to different plant toxic metabolites and pesticides (Agut et al., 2018; Santamaria et al., 2019). Secondary metabolite biosynthesis, such as that of glucosinolates and JA-dependent responses, are important components of the defense of *Arabidopsis* against mites (Zhurov et al., 2014). However, rearing mites on *Arabidopsis* hosts for multiple generations results in rapid adaptation to the plant’s defenses. Plant survival likelihood increases when defense mechanisms are based on a wide range of molecular and chemical responses. In this regard, the resistance of citrus plants to *T. urticae* is more effective when both antibiosis and antixenosis are activated rapidly after infestation (Agut et al., 2014, 2015). JA-dependent signaling is effective in *Citrus aurantium* (sour orange) counteracting *T. urticae*, however, negative SA-JA crosstalk is non-functional in this case and may contribute to bypass mite manipulation of the host, as observed in the more susceptible genotype *C. reshni* (Agut et al., 2014). Additionally, following herbivory, citrus plants release a plethora of volatile organic compounds that exert additional antixenotic effects against mites (Agut et al., 2015) and attract *T. urticae* natural enemies (indirect defense) (Cabedo-López et al., 2019). *C. aurantium* is more resistant than other citrus rootstocks (Bruessow et al., 2010); moreover, *C. aurantium* infested with two-spotted spider mites shows a rapid increase in the phytohormone JA and accumulates three phenolic derivatives, (i.e., 4-coumaric acid, naringenin, and hesperetin) together with putrescine and tryptophan, all of which show priming profiles, compared to the susceptible genotype *C. reshni* (Agut et al., 2014). When *C. aurantium* perceives herbivory, a systemic signal that involves Glu rapidly activates resistance in systemic tissues in a very efficient manner, thereby eliciting systemic resistance (Agut et al., 2016; De Kesel et al., 2021). Of note, Glu signaling depends on JA increases in locally infested tissues (Qiu et al., 2020).

Citrus plants are among the 80% of mycotrophic plant species that are well colonized by fungi of the division Glomeromycota. Unlike tomato plants, roots of citrus plants can be successfully colonized during full phosphorus fertilization (Ortas, 2012). Most studies on symbioses of citrus focused on the improvement of tolerance to abiotic stress (He et al., 2020; Hadian-Deljou et al., 2020). However, there are few reports on immunity in mycorrhized citrus plants. Moreover, very few studies described improved defense mechanisms in mycorrhized citrus trees under biotic stress. Some studies suggested that mycorrhized citrus can better resist infection with citrus canker *Xantomonas axonopodis* (Xie et al., 2019). Mycorrhized *Poncirus trifoliata* rapidly activates SA-dependent signaling, such as through the genes *PtEPS1* and *PtPR4*, thus ameliorating adverse effects of infection such as H_2_O_2_ reduction and SA suppression by bacteria (Xie et al., 2019). Infected mycorrhized plants respond by enhancing SA signaling, which is transferred through common mycorrhizal networks to neighboring plants (Zhang et al., 2019). It was suggested that either SA or the SA signal is transported through common mycorrhizal networks, thereby stimulating higher levels of Pathogenesis-related (PR) protein, such as PtPR1, PtPR4, PtPR5, and PtNPR1. Studies on mycorrhiza-induced resistance (MIR) against the oomycete *Phytophthora parasitica* revealed that mycorrhized plants show priming of SA-dependent responses, resulting in enhanced resistance (Tian et al., 2021). These studies suggest that SA is stimulated in mycorrhized plants downstream of *PtMAPK3*. In tomato plants, colonization with arbuscular mycorrhizal fungi (AMF) elicits JA-dependent responses that promote resistance against necrotrophic pathogens and herbivorous insects such as *Spodoptera exigua* (Lepidoptera: Noctuidae) (Song et al., 2013; Rivero et al., 2021). In fact, plants colonized with AMF show enhanced resistance to the leaf-chewing insect *Helicoverpa arimigera* (Lepidoptera: Noctuidae); mycorrhizal symbiosis helps reduce herbivory as it interferes with the performance of caterpillars which then show reduced larval weight. Mycorrhized plants exhibit priming of the JA-dependent genes *LOXD, AOC, PINI*, and *PINII* in a time-dependent manner (Song et al., 2013). JA-mediated MIR is further supported by the fact that *spr2* and *jai1* do not display enhanced resistance (Song et al., 2013; Rivero et al., 2021). Evidence suggests induction of SA-dependent defenses in citrus during defense against *X. axonopodis* and *P. parasitica*, indicating that priming may be the main mechanism underlying MIR, as priming is a horizontal phenomenon that can activate different defenses, depending on the pathosystem (Mauch-Mani et al., 2017).

Despite these interesting findings, no studies examined defense mechanisms of mycorrhized citrus against spider mites. Hoffmann et al. (2011a) suggested that mycorrhizal bean plants experience increased oviposition by the two-spotted spider mite *T. urticae* due to increased host quality; however, this apparent increase of susceptibility is compensated by increased attraction of the pest’s natural enemy *Phytoseiulus persimilis* (Acari: Mesostigmata), resulting in reduced pest populations on bean plants colonized with *Funneliformis mosseae* (Hoffmann et al., 2011a,b). Similar observations were made using the endophytic fungus *Fusarium solani* strain K, which led to increased plant biomass during mite infestation and also resulted in enhanced attraction of the generalist predator *Macrolophus pygmaeus* (Pappas et al., 2018). Colonization by the fungus resulted in significant changes in volatile compounds, which was suggested to modify the behavior of predator bugs. In conclusion, mycorrhizal symbiosis alters physiological and molecular plant parameters which modulate aboveground associations and tri-trophic interactions, resulting in a marked benefit for the plant partner.

In the present study, we characterized mycorrhized *C. aurantium* responses against the generalist adapted pest *T. urticae* feeding on *Citrus clementine cv* clemenules. Damage rates and mite performance were recorded, and we characterized local and systemic responses to infestation. Moreover, we conducted preference assays with mites allowed to choose between mycorrhized and non-mycorrhized plants. Our results provide evidence of the benefits of mycorrhizal symbiosis regarding resistance against polyphagous and cosmopolitan pests which are difficult to control.

## Materials and Methods

### Plant Material and Mycorrhizal Colonization

*Rhizophagus irregularis* (formerly *Glomus intraradices*) was maintained in clover roots until plant inoculation. *Citrus aurantium* seeds were germinated and grown in vermiculite and peat (1:3 v/v) in a climate chamber with a 16:8 h light-dark photoperiod at 24°C day and 18°C night temperature and 50–70% relative humidity. Two-months-old citrus seedlings were transplanted to 320-cm^3^ pots containing sterile vermiculite and 15% (v/v) of mycorrhizal inoculum. Plants used as controls were transplanted to 320 mL pots containing sterile vermiculite and were irrigated using aliquots of the inoculum filtrate to replicate the microbiome. One month after inoculation, random samples of 4 to 5 mycorrhized citrus plant roots were collected and were stained using the Vierheilig method (Vierheilig et al., 1998). Roots were cut into 2 cm segments, and the proportion of total root colonization was measured using the gridline intersection method (Giovannetti and Mosse, 1980) with a Nikon Eclipse 50i microscope under bright-field conditions to ensure that the plants had reached an adequate level of mycorrhization. We examined 150–200 sections per assay. Plants were irrigated every three days using Hoagland’s modified solution (Hoagland and Arnon, 1950), and these plants were not treated with any pesticides, acaricides, or fungicides.

### Spider Mite Stock Colony

The polyphagous mite *T. urticae* was used for performance assays. The *T. urticae* colony was started in Castelló de la Plana, Spain, and was maintained on lemon (*C. limon* (L.)) fruits obtained from a pesticide-free orchard at Universitat Jaume I under a natural photoperiod at 25°C and 50–70% humidity. Lemons were replaced every two to three weeks.

When cohorts were required, females of the same stage and age were obtained by transferring gravid females from the stock colony to detached citrus Clementina (*C. reshni*) leaf units. These leaves were placed upside-down on sponges covered with wet cotton which were placed on top of a plastic tray that was half-filled with water to prevent mite dispersal and to maintain moisture. Two to three days later, females were removed using a paintbrush, and eggs were maintained in the detached leaves for 10–13 days until they reached the target stage and age for use in the experiments. Adult females used for assays were starved for 24 h before experiments.

### *Citrus aurantium* Local Defense Against *Tetranychus urticae*

Assays were carried out in a climate chamber under the same conditions as described above to prevent mite dispersal. To determine damage and oviposition rates by *T. urticae*, 12 plants were used per repetition. Half of the plants were arbuscular mycorrhized (AM) and the other part was used as a control plant (NM). The experiment was repeated three times with similar results. Before infestation, the lower part of the stem and infested leaves of each plant were covered with Vaseline. The proportion of infestation was two leaves per plant with five 6-days-old female mites from the cohort described above. Leaves were detached to count the number of adults and eggs per each leaf using a binocular microscope three days post-infection. Leaves were then cleaned and scanned to estimate the damaged leaf surface using GIMP software (GNU Image Manipulation Program v 2.10.20). Plant material (2 infested leaves per plant) at 24 and 48 h post-infection (hpi) was collected and frozen at –80°C for gene expression quantification and metabolomic analyses.

### Systemic Resistance Assays of AM *Citrus aurantium* Infested With *Tetranychus urticae*

Experiments were carried out under the same conditions as described above. 24 plants were employed per repetition. 50% of the total plants were mycorrhized while the other half were used as NM. The experiment was repeated three times with similar results. The lower and the medium parts of the stem of each plant were covered with Vaseline to separate local and systemic leaves and prevent mite dispersal. Half of the control and AM plants were randomly infested locally on leaves using 10 *T. urticae* females. Three days after the first infestation, two distal leaves of each plant were infested using five mites per leaf. Three days after this, leaves of systemically infected plants were detached and scanned to estimate the damaged leaf surface using GIMP 2.10 software.

### Olfactometer Assays

Assays were carried out using a Y-tube as described by Bruin et al. (1992). We used a 4-cm diameter glass tube with two arms of 13.5 cm long containing in the core of the olfactometer a Y-shaped metal wire of 1 mm diameter with the same length as the Y-tube. The two arms were connected with two 5-L glass vessels through plastic pipes. Each vessel was connected to a pump that produced a unidirectional air flow of 1.5 LPM from the arms to the end of the Y-tube. The air was continuously purified using an active charcoal filter (Sigma-Aldrich, Spain). The environmental conditions were 25°C and 50–70% relative humidity.

The olfactometer was cleaned using acetone one day before each assay. One plant per condition was placed in a vessel 30 min before the experiment. The combinations for antixenosis assays were as follow: (a) non-mycorrhizal non-infested plants (NM) versus non-mycorrhizal infested plants (NM inf); (b) non-mycorrhizal NM versus mycorrhizal non-infested plants (AM); (c) mycorrhizal non-infested plants (AM) versus mycorrhizal infested plants (AM inf); and (d) non-mycorrhizal infested plants (NM inf) versus mycorrhizal infested plants (AM inf). NM and AM plants without infestation were also stained with vaseline to avoid external responses. Adult females from the lemon stock colony were used to infest *C. aurantium* plants and were starved for 24 h before the two-choice assay. For the assays in which the spider mites must choose among plants already infested (AM inf, NM inf, in whatever combination) those plants were infested 48 h before the choice assay. A female mite was placed at the beginning of the metal wire and was allowed to choose within 5 min between two conditions. Spider mites that did not reach the end of one of the arms of the wire within the time of the experiment were scored as no-choice. After every 15 mites, the system was cleaned, and the plants were exchanged. At least 45 females with a clear choice and four plants per combination were used.

### RNA Extraction and Quantitative Real-Time PCR Analysis

RNA extraction from leaves was carried out as described by Kiefer et al. (2000), with some modifications. Trizol (1 mL) was added to 90 mg fresh ground leaves. The extraction was performed by sampling the two infested leaves from six plants per condition, with a total of n = 3. For the RNA extraction three biological replicates were prepared. The samples were incubated for at least 5 min at room temperature. After centrifugation, the supernatant was transferred to an Eppendorf tube containing 220 μL CHCl_3_. Samples were centrifuged, and the supernatant was placed in a new tube; 350 μL isopropanol and 350 μL of a mix of 0.8 M citrate and 1.2 mM NaCl were added. After centrifugation, the supernatant was removed, and the pellet was rinsed twice and centrifuged each time with 70% EtOH (0.5 mL). After the last centrifugation, the pellet was air-dried and dissolved in 25 μL nuclease-free water. The sample was stored at –20°C, and one day later, RNA was measured using a Nanodrop device to adjust the concentration to 1 μg/mL. The samples were treated with DNase (Thermo Fisher Scientific) to remove contamination. cDNA was synthesized using a High-Capacity cDNA Reverse Transcription Kit (Applied Biosystems). Quantitative real-time PCR was performed using AceQ Universal SYBR Green qPCR Master Mix (Vazyme) using a StepOne instrument (Applied Biosystems). Primers for *PR-5*, *LOX2*, *PR-3*, and *CHS* were used (Agut et al., 2014). *Elongation factor 1-alpha* (*EF1-*α) and *Ubiquitin Protein Ligase 7* (*UPL7*) were used as housekeeping genes to normalize the results. *EF1-*α primer sequences were published by Agut et al. (2014) and those of *UPL7* primers by Mafra et al. (2012).

### Metabolome Analysis and LC-ESI Full Scan Mass Spectrometry Q-TOF

Samples for metabolomic analysis were performed by sampling the two infested leaves from 6 plants per condition and experiment. A total of three biological replicates and two techniques for three experiments were used for this analysis (n = 5). Metabolites were extracted using a mixture of MeOH/H_2_O (30:70) supplemented with 0.01% HCOOH. Then, 1 mL MeOH 30% was added to 300 mg powdered freeze-dried tissue. The supernatant was filtered using a 0.2 μ cellulose filter (Phenomenex). An aliquot of 20 μL of each sample was injected into the UPLC in positive (ESI+) and negative (ESI-) ion modes for electrospray ionization. A reversed-phase Acquity UPLC system (Waters, Milford, MA, United States) with a gradient of MeOH and H_2_O supplemented with 0.01% HCOOH coupled to a hybrid quadrupole time-of-flight instrument (QTOF MS Premier) was used to elute and detect metabolites. A second fragmentation function was used to identify the signals, as described by Sanchez-Bel et al. (2018). Two internal libraries were used for metabolite identification. Both libraries were created using pure chemical standards to record the specific retention time, exact mass, and spectrum fragmentation, as described in Gamir et al. (2014) and Schymanski et al. (2014). Compounds without standard in the libraries were identified using the online databases Metlin,^1^, PubChem,^2^ or Massbank^3^ to compare their fragmentation spectra.

### Antibiotic Properties of Phenylpropanoids Derivative Compounds in Citrus Plants

Non-mycorrhizal *C. aurantium* plants were sprayed with six different compounds (kaempferol, luteolin, diconyferil alcohol, neohesperidin, malic acid, cinnamic acid) at a concentration of 0.5 mM in the range of other treatments with flavonoids (Wachter et al., 1999; Stec et al., 2021). Treatments were grouped according to the solvent used as a control. DMSO at a final concentration of 1% was used as a solvent for kaempferol, luteolin, diconyferil alcohol, and neohesperidin; 1% DMSO 1% with no other chemical was used for the control treatment. For malic acid and cinnamic acid, water was used as a solvent; the controls were treated with water only. All treatment solutions contained 0.01% TWEEN-20 as a wetting agent. Plants were infested with five mites per leaf on two leaves per plant, 24 h after the treatments. Seven days after infestation, the infested leaves were detached and scanned to estimate the damaged leaf surface, as described above. The environmental conditions were 25°C and 50–70% relative humidity. Three plants were used for each treatment and the experiment was repeated three times.

### Full-Scan Data

Raw data from Masslynx 4.2 (Masslynx 4.2, Waters) software were transformed into.cdf format using the Masslynx Databridge tool. To process the data, R software v. 4.0.3 was used to separately analyze ESI + and ESI- signals. Signal corrections were obtained using the XCSM algorithm^4^ for R. The amount of each compound was determined from the normalized peak area units relative to the dry weight of each sample. Signals of different treatments were compared using the Kruskal–Wallis test (*P* < 0.05) following adduct and isotope correction. Mar-Vis Suit 2.0 was used to obtain isotope corrections, clustering, heatmaps (Mar-Vis Cluster), and pathways (Mar-Vis pathway) with different changes within treatments. MetaboAnalyst 4.0 was used for principal component analysis (PCA) applying normalization by median followed by cube root transformation and Pareto scaling. Raw data are available in Supplementary Table 2.

### Statistical Analysis

In order to perform the proper statistical analysis, normality and homoscedasticity of the data were previously analyzed. In the case of the hormones and flavonoids quantification, data fulfill normality assumption, but not homoscedasticity, thus a Two-Way ANOVA test was used to evaluate two categorical variables: mycorrhization and infestation. For significant interactions among factors, a Tukey test was performed. For gene expression, phenylpropanoid treatments and systemic phenotype analysis Generalized Linear Model (GLIM) statistics were performed, since the data don’t fulfill normality and homoscedasticity, comparing each treatment to the control (NM). Relative leaf damaged area and oviposition were compared using *t*-test. Olfactometer assays were contrasted using a binomial test from a 50:50 distribution. SPSS 25.O (SPSS Statistics, SPSS Inc., Chicago, IL, United States) was employed to carry out all statistical analyses (GLM, GLIM, *t*-test, binomial test). Statistical significances for all tests are provided in Supplementary Table 1.

## Results

### Mycorrhizal Symbiosis Increases Resistance of *Citrus aurantium* Against Spider Mites but Does Not Affect Host Choice of Mites

**FIGURE 1:**
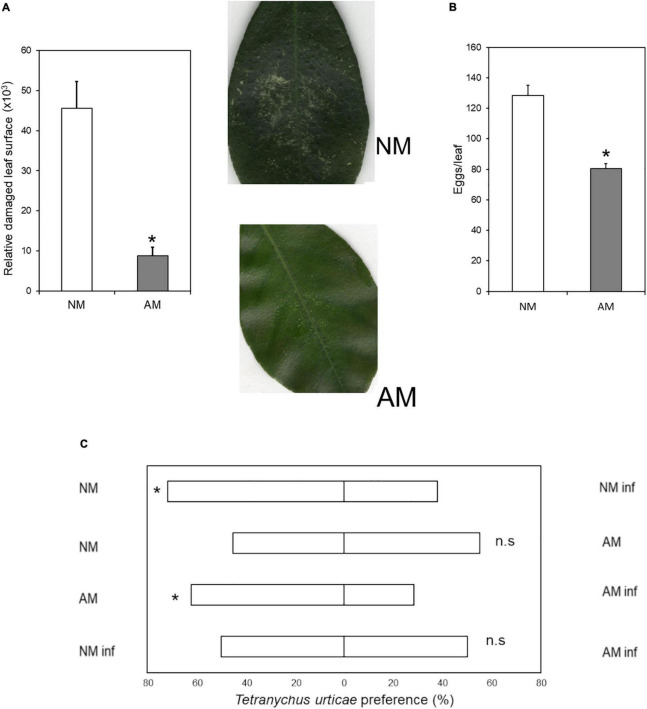
Antibiosis and antixenosis of *C. aurantium* NM and AM plants upon *T. urticae* infestation. **(A)** Relative leaf damaged area: Three-months-old plants were infested with 5 mites per leaf (two leaves per plant). Asterisks indicate significant differences between treatments (*P* < 0.05; *t*-test; n = 6). Pictures show an example of the damage. **(B)** Oviposition in mycorrhizal and non-mycorrhizal *C. aurantium* plants: 3-month-old *C. aurantium* plants were infested with 2-days-old *T. urticae* females (5 females per leaf, two leaves per plant). The number of eggs was determined 3 days after infestation. Different letters indicate significant differences between treatments (*P* < 0.05; *t*-test; n = 6). **(C)** Antixenotic effects on *T. urticae* performance when exposed to odors of mycorrhizal or non-mycorrhizal plants or plants previously infested with conspecifics. Four different assays were carried out where mites had to choose between plant odors of two different treatments. A minimum of 4 plants and 45 spider mites were tested for each combination. Every assay was repeated a minimum of three times on different days. The different combinations performed, were as follows: First: non-mycorrhizal non-infested plants (NM) versus non-mycorrhizal infested plants (NM inf); second: non-mycorrhizal non-infested plants (NM) versus mycorrhizal non-infested plants (AM); third mycorrhizal non-infested plants (AM) versus mycorrhizal infested plants (AM inf) and fourth: non-mycorrhizal infested plants (NM inf) versus mycorrhizal infested plants (AM inf). Plants were randomly infested with 20 adult females 24 h before assays. Mites used to choose between combinations were starved 24 h before the assays. Asterisks indicate significant differences for a 50:50 distribution (binomial test; *P* < 0.05).

To understand whether symbiosis between *C. aurantium* and *R. irregularis* modifies aboveground defenses against mites, we studied the performance of *T. urticae* in three-month-old seedlings. Sixty-days-old plants were inoculated with *R. irregularis*, and 30 days later, randomly selected plants were harvested to assess colonization. In all cases, citrus roots presented levels of colonization above 25–35% (Supplementary Figure 1). NM and AM plants were infested with 10 mites per plant. Symbiosis resulted in reduced mite performance (Figure 1). AM *C. aurantium* plants exhibited lower leaf damage (Figure 1A). The number of eggs per leaf was reduced by 30% although the number of alive mites did not change at 72 hpi (Figure 1B). As a component of resistance, we also tested potential antixenotic effects of AMF colonization. The mites were allowed to choose between AM and NM citrus seedlings in Y-tube assays (Figure 1C). Mites did not choose between NM and AM control plants; however, when plants were subjected to herbivory for 48 h, the plant odor was clearly repellent, independent of AMF colonization. Seemingly, mites did not choose between pre-infested NM and AM plants, suggesting that deterrency on mite performance in AM plants is due to direct intracellular defenses rather than due to volatile compounds.

### Hormonal Regulation of AM Citrus in Response to *Tetranychus urticae* Herbivory

Phytohormone-based immune responses against spider mites have been reported previously (Song et al., 2013; Agut et al., 2014; Santamaria et al., 2020); we therefore analyzed expression of SA-and JA-dependent genes at 24 and 48 hpi. In addition, we searched for the phytohormones SA, OPDA, JA, and JA-Ile in the metabolomic data sheet at 24 hpi through non-targeted analysis. Subsequently, these signals were confirmed by comparing retention times and spectrum fragmentation using an internal library of chemical standards (Gamir et al., 2014; Schymanski et al., 2014).

**FIGURE 2:**
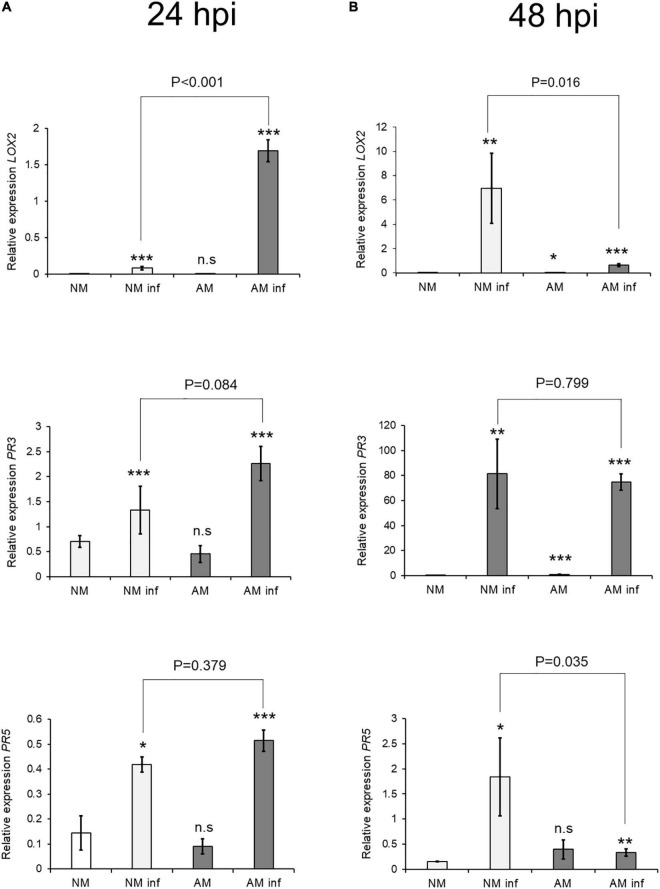
Effect of mycorrhization on Defense-Related Gene Expression 24 h after *T. urticae* infestation. Gene expression transcript levels at **(A)** 24 h after infestation and **(B)** 48 h post infestation of the oxylipin biosynthesis (*LOX2*) and signaling (*PR3*) JA-related genes and a signaling (*PR5*) SA-related gene were normalized to the expression of the constitutive gene *UPL7*, measured in the same sample. Three treatments: AM: mycorrhizal non-infested plants. NM inf: non-mycorrhizal infested plants. AM inf: mycorrhizal infested plants, were compared to the control (NM: non-mycorrhizal non-infested plants). Asterisks indicate significant differences at: *P < 0.05, **P < 0.01, ***P < 0.001 for generalized linear model (GLIM, n = 3). AM and AM inf were also compared. AM and AMinf were also compared using GLIM and the *P*-value is presented on the figure. Three independent assays were carried out with similar results.

The biosynthesis gene *LOX2* and the marker gene *PR3*, which are involved in the oxylipin-mediated defense responses, showed a priming profile at 24 hpi in AM plants (Figure 2). The SA marker gene PR5 was also induced after infection, as observed previously by Agut et al. (2014). Symbiosis elicited upregulation of *PR5* gene expression in infected plants, indicating that SA-JA crosstalk was deregulated in *C. aurantium*. At 48 hpi, expression of *LOX2* and *PR3* increased in NM plants after infestation, indicating faster responses in AM than in NM plants.

**FIGURE 3:**
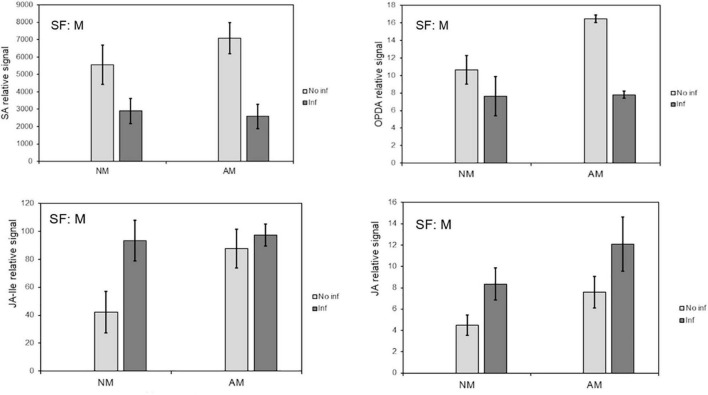
Influence of mycorrhization on phytohormones that regulate plant defence against *T. urticae* infestation. Content of the defense-related phytohormones OPDA, SA, Me-JA and JA in local leaves from mycorrhizal and non-mycorrhizal *C. aurantium* plants at 48 h post infestation. The levels were obtained from the non-targeted metabolome analysis by UPL-QTOFMS. Four treatments were compared: NM: non-mycorrhizal non-infested plants. AM: mycorrhizal non-infested plants. NM inf: non-mycorrhizal infested plants. AM inf: mycorrhizal infested plants. Three independent and two technical replicates were randomly performed. Significant factors (SF) indicate if the two independent factors: A (Arbuscular Mycorrhizal), M (Mite infestation) and/or their interaction I (A × M) were statistically significant (Two-way ANOVA, *P* < 0.05). The stats of analysis are provided in Supplementary Table 1. Data was presented as a mean of five replicates ± SD (n = 5).

Hormone analysis revealed that infestation reduced SA levels, irrespective of AMF colonization (Figure 3). JA and JA-Ile concentrations were increased in NM plants in response to mite infestation. All tested oxylipins increased in AM plants, whereas JA and JA-Ile remained elevated following infestation. This may suggest a better preparation of mycorrhizal plants before infestation, during which OPDA is increased.

### AMF Colonization Strongly Affects the Metabolomic Profile of *Citrus aurantium* Plants

**FIGURE 4:**
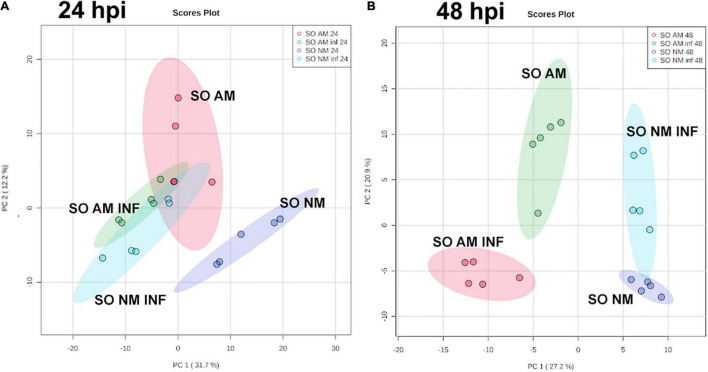
Impact of mycorrhization on the metabolic profile of locally infested leaves of *C. aurantium* plants. Non-targeted principal component analysis (PCA) obtained from a non-target analysis by UPLC-QTOFMS of locally infested leaves at **(A)** 24 h post infestation and **(B)** 48 h post infestation with *T. urticae.* 3 months old plants were infested with five 2-days-old female *T. urticae* (2 leaves per plant). 24 or 48 h after infestation, infested leaves were detached and frozen at –80°C. A pool from 6 individual plants was employed for each combination. Three independent and two technical replicates were randomly performed (n = 5). Four different treatments were analyzed: NM: non-mycorrhizal non-infested plants. AM: mycorrhizal non-infested plants. NM inf: non-mycorrhizal infested plants. AM inf: mycorrhizal infested plants.

**FIGURE 5:**
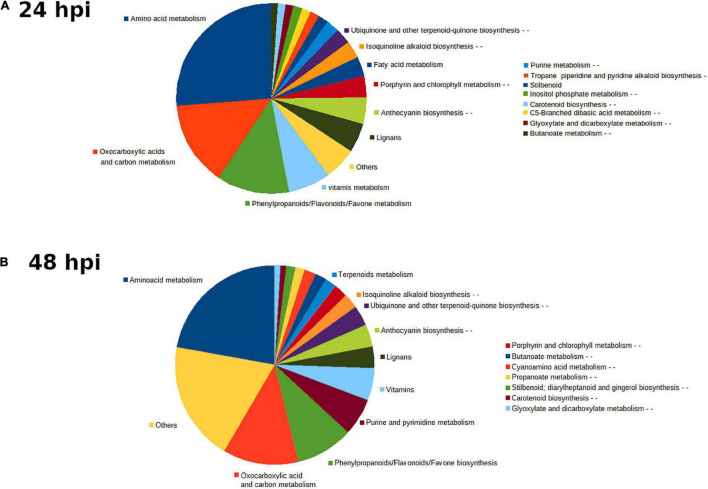
Main pathways implicated in *C. aurantium* response upon *T. urticae* infestation. Sector graph of from mycorrhizal and non-mycorrhizal *C. aurantium* plants or plants previously infested with *T. urticae* obtained from the non-targeted metabolomic analysis by UPLC-QTOFMS. Pathways with signals more accumulated of AM inf-AM locally infested leaves compared with NM inf-NM at **(A)** 24 h and **(B)** 48 h after infestation. Four different treatments were employed: NM: non-mycorrhizal non-infested plants. AM: mycorrhizal non-infested plants. NM inf: non-mycorrhizal infested plants. AM inf: mycorrhizal infested plants. Pathways with more changes were obtained by the software MarVis (Marvis Pathway). Signals from different treatments were compared after a Kruskal–Wallis test (*P* < 0.05) following adduct and isotope correction. A pool from 6 individual plants was employed for each combination. Three independent and two technical replicates were randomly performed (n = 5).

**FIGURE 6:**
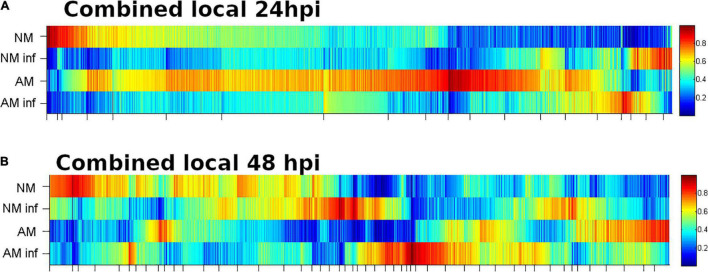
Heatmap analysis of locally infested *C. aurantium* leaves. Results from mycorrhizal and non-mycorrhizal non-infested *C. aurantium* plants or plants previously infested with *T. urticae* at **(A)** 24 h or **(B)** 48 h post infestation. Four different treatments were employed in both cases: NM: non-mycorrhizal SO plants. AM: mycorrhizal SO non-infested plants. NM inf: non-mycorrhizal infested plants. AM inf: mycorrhizal infested plants. Heatmaps were obtained by the software MarVis (Marvis Cluster). Signals of different treatments were compared after a Kruskal–Wallis test (*P* < 0.05) following adduct and isotope correction. A pool from 6 individual plants was employed for each combination. Three independent and two technical replicates were randomly performed.

To understand the molecular responses of AM citrus plants to infestation, we determined metabolomic profiles at early time points such as 24 hpi, where injuries were hardly visible, and at 48 hpi, when the damage produced by mites was evident. *C. aurantium* plants responded rapidly to infestation by a strong metabolomic rearrangement at 24 hpi (Figure 4A). Unsupervised PCA showed that infested *C. aurantium* plants were grouped separately from non-infested plants. Thirty days after root inoculation with *R. irregularis*, symbiosis elicited metabolomic changes in the leaves, which was mostly shown by the second component PC2 explaining 12.2% of the differences, and AM plants were segregated from NM control plants. These changes were less pronounced when PC1 corresponded to 31.7% of the changes. Following infestation, infested AM and NM plants partially overlapped at 24 hpi, indicating a reduced impact of mycorrhization following infestation at early time points (Figure 4A). At 48 hpi, PCA showed that all groups of treatments behaved differently, and AM plants were segregated from non-infested AM plants (Figure 4B). We performed a pathway analysis using MarVis software after isotope and adduct correction of ions. Using tentative IDs based on exact mass, we selected signals accumulated in AMinf-AM plants compared with NMinf-NM plants for pathway classification. At 24 hpi, the main changes were observed in the primary metabolism, and amino acid metabolism and oxocarboxylic acids comprised more than 40% of signals accumulated in AMinf/AM plants compared with NMinf/NM controls (Figure 5). At this time point, phenylpropanoids and vitamins already represented approximately 19% of the signals, indicating an impact in the secondary metabolism due to symbiosis. At 48 hpi, a reduction in the primary metabolites, such as amino acids and oxocarboxylic acid, and carbon metabolism (approximately 33%) occurred, whereas the number of hits of other pathways of the secondary metabolism increased. This switch may suggest an increase in defensive compounds. This hypothesis was further supported by heatmap analysis (Figure 6). At 24 hpi, AM plants showed the most pronounced differences from NM plants (Figure 6A). At 24 hpi, infestation elicited suppression of most signals that were less accumulated compared to non-infested plants, which is indicative of host defense hijacking. Infested AM plants reacted strongly at 48 hpi (Figure 6B). This was confirmed by an increase in accumulated signals in infected AM plants. Many of these signals displayed priming profiles, whereas only few compounds were primed at 24 hpi. By selecting hits within the pathways of the oxocarboxylic acids and phenylpropanoids (Supplementary Figure 2), an increase in priming compounds at 48 hpi was observed, which may be indicative of a more robust defense response in locally infested leaves of AM plants.

**FIGURE 7:**
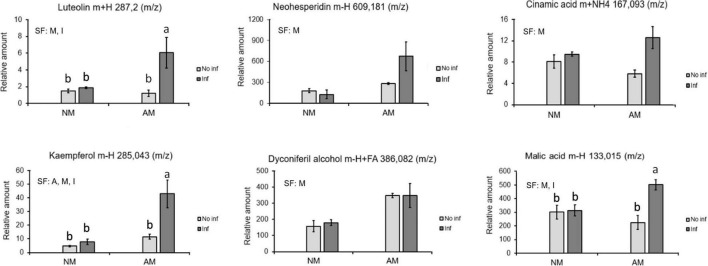
Identified compounds displaying a priming profile in mycorrhizal *C. aurantium* plants 48 h after *T. urticae* infestation. Four different treatments were employed in all the cases: NM: non-mycorrhizal SO plants. AM: mycorrhizal SO non-infested plants. NM inf: non-mycorrhizal infested plants. AM inf: mycorrhizal infested plants. Compounds were identified using exact mass and fragmentation spectrum. Peak intensity was obtained by using MarVis filter and cluster following a Kruskal–Wallis test (*P* < 0.05). Relative amount of the metabolites was determined by normalizing the chromatographic area of each compound with the dry weight of its sample. Data was presented as a mean of five replicates ± SD (n = 5). Significant factors (SF) indicate if the two independent factors: A (Arbuscular Mycorrhizal), M (Mite infestation) and/or their interaction I (A × M) were statistically significant (Two-way ANOVA, *P* < 0.05). Different letters indicate significative differences (Tukey HSD test, *P* < 0.05). The stats of analysis are provided in Supplementary Table 1.

Focusing attention on the priming clusters at 48 hpi, several phenolic derivative compounds such as luteolin, neohesperidin, kaempferol, diconiferyl alcohol, cinnamic acid, oxocarboxylic acid, and malic acid were identified (Figure 7).

### Functional Characterization of Priming Compounds

**FIGURE 8:**
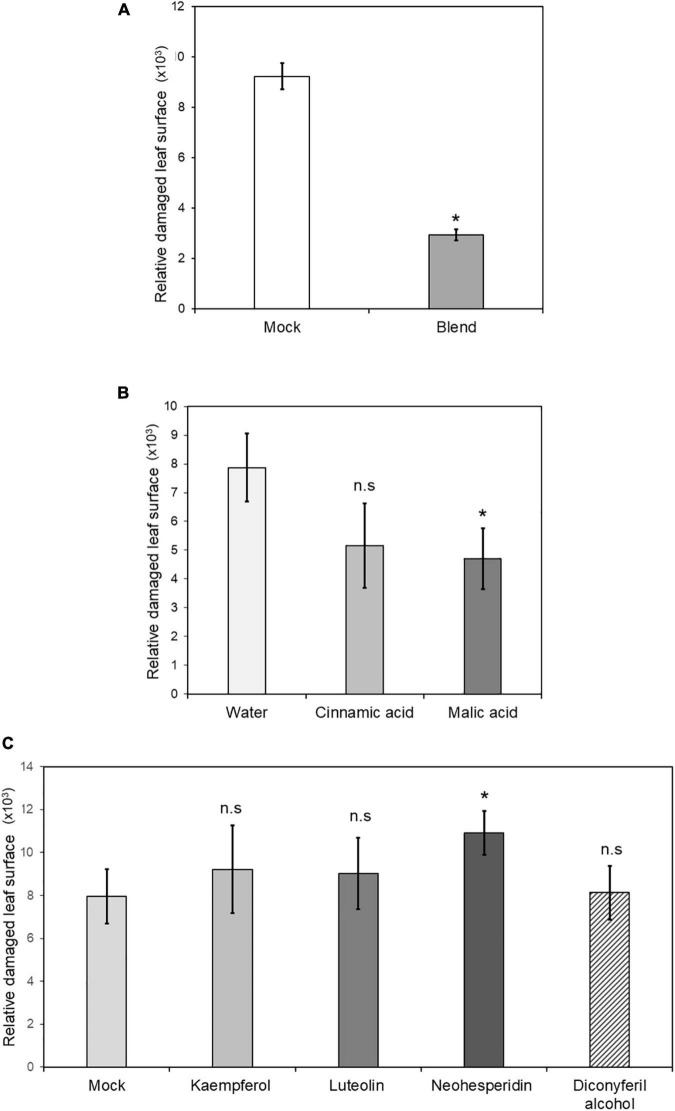
Induced resistance by chemical treatments of priming compounds. Four non-mycorrhizal C. *aurantium* plants were infested with 5 *T. urticae* per leaf (two leaves per plant) after foliar application with either malic acid, cinnamic acid, kaempferol, luteolin, diconyferil alcohol, neohesperidin or a blend of all of them. Treatments were grouped depending on the control **(A)** 1% DMSO, **(B)**, water **(C)** 1% DMSO. Infested leaves were detached and scanned to estimate damaged leaf surface as described above. The experiment was repeated three times. Asterisks indicate significant differences at *P* < 0.05 (GLIM, n = 3).

To determine the relevance of the secondary metabolites identified in MIR against *T. urticae*, functional tests of induced resistance were performed by spray application of these compounds, either in single solutions or in a mixture containing all of them at two different concentrations. Neither malic acid, single phenolics, nor flavonoids at 0.5 mM reduced mite-induced damage in the leaves (Figure 8). The combination of all compounds in a mixture resulted in strong protection and reduced the damage rate by 80% when applied.

### Systemic ResIstance of *Citrus aurantium* Against *Tetranychus urticae* Is Not Affected by Mycorrhization

Previous studies indicated that sour orange plants show systemic resistance 72 h after mite infestation (Agut et al., 2016), hence systemic tissues are more resistant. Considering the enhanced resistance observed in locally infested leaves of AM plants, we hypothesized that MIR may also be observed in systemic tissues. PCA of the metabolomic changes in systemic leaves showed that at 24 hpi, the changes in AM plants were segregated from those in NM control plants observed in locally non-infested leaves. However, after infestation, the metabolomic response merged irrespective of mycorrhization (Supplementary Figure 3), suggesting a similar systemic response to mite infestation in NM and AM distal leaves. At 48 hpi, all groups merged, indicating a minimal impact on systemic leaves of a second infestation and the symbiosis, which is indicative of a strongly reduced impact of mite herbivory on distal leaves (Supplementary Figure 3). According to pathway analysis of the signals accumulated in AM-AMinf vs. NM-NMinf systemic leaves, at 24 hpi, amino acid metabolism, oxocarboxylic acids, and terpenoids were among the main groups of compounds (Supplementary Figure 4). At 48 hpi, there was a strong change in the main pathways displaying the highest number of signals because the pathway of lignans was highly represented, whereas the amino acid pathway was strongly reduced compared with early time points (Supplementary Figure 4).

Changes in the composition of systemic metabolites and time dynamics likely influenced the response of distal leaves of AM plants. To test whether AM plants would show higher levels of systemic resistance, systemic leaves from infested NM and AM plants were tested for resistance. The first infestation with 10 mites per leaf was restricted to the bottom leaves using Vaseline. A second infestation 72 h later elicited a strong systemic resistance in distal leaves, reducing the damage rate by 86% (Supplementary Figure 5). Despite the expected change in systemic resistance, AM plants displayed the same level of enhanced systemic resistance as NM plants, probably due to the strong level of response in NM *C. aurantium* plants (Supplementary Figure 5). These observations suggest that metabolomic changes in systemic tissues do not improve basal systemic resistance.

## Discussion

In the present study, we confirmed that MIR is functional in citrus defense against adapted lines of *T. urticae* grown for several generations on lemon as a neutral host. Unlike several studies indicating that mite performance is improved in mycorrhized plants such as common bean (Hoffmann and Schausberger, 2012; Patiño-Ruiz and Schausberger, 2014), *C. aurantium* plants displayed reduced damaged leaf surface and oviposition after three days of herbivory. Notably, *C. aurantium* has a strong basal level of resistance against this mite compared to other citrus genotypes (Bruessow et al., 2010; Agut et al., 2014).

Among the two main resistance traits of resistance, antibiosis and antixenosis, as described in *C. aurantium* defense against the two-spotted spider mites, MIR seems to be functionally reinforcing antibiosis but not antixenosis, as mites did not show a preference for AM or NM plants. In addition, we identified priming compounds that likely mediate MIR in citrus plants. Approaches to determine whether symbiosis contributes to enhanced expression of systemic resistance were also implemented. In this study, we did not find differences with NM plants when assessing damage rates in systemic leaves, which may be due to the already strong systemic resistance in non-mycorrhized citrus.

The functionality of MIR in citrus has been confirmed in several studies regarding fungal, bacterial, and oomycete pathogens. Mycorrhizal symbiosis enhances citrus resistance against *X. axonopodis* by stimulating SA-dependent responses, such as PR1, PR5, and NPR1, together with increased SA levels (Zhang et al., 2019). Similarly, mycorrhized *P. trifoliata* accumulates higher levels of SA and shows increased PAL1 and EPS1 gene expression regardless of *P. parasitica* infection (Tian et al., 2021), which contributed to MIR against the oomycete in citrus plants. However, there is a lack of studies on mycorrhized citrus infested by the polyphagous mite *T. urticae*. Defense against spider mites remains under intense focus of study because *T. urticae* can efficiently evade insecticidal proteins and chemical plant defenses (Santamaría et al., 2015; Santamaria et al., 2020) which provides a great adaptability to pesticides. The adaptation of the mite to the host strongly determines the final outcome of this herbivory (Blaazer et al., 2018).

In addition to mite adaptation, the complexity of mite-citrus interaction is further conditioned by the host genotype. Bruessow et al. (2010) characterized a range of citrus genotypes with remarkable differences in mite resistance. Subsequent studies on the most distant genotypes *C. aurantium* (SO comparably resistant) and *Cleopatra* mandarin (*C. reshni*; very susceptible) concluded that herbivory in these two cultivars is mostly determined by two main factors, i.e., absence of negative crosstalk SA-JA in *C. aurantium*, which contributes to faster and stronger accumulation of JA-dependent defenses effective against the mite (Agut et al., 2014.), and accumulation of secondary metabolites such as flavonoids, polyamines, and alkaloids that contribute to antibiosis in locally infested leaves (Agut et al., 2014, 2015).

In the present study, we determined that MIR further protected *C. aurantium*, eliciting enhanced resistance by increasing antibiosis. Both infested NM and AM plants showed many down-regulated compounds at 24 h after infestation. This suggests that mites suppressed the plants’ early metabolic responses; however, at 48 hpi, infested AM plants displayed a higher number of over-accumulated metabolites, which may contribute to the enhanced resistance observed in mycorrhizal plants. In addition, as observed in AM plants, the metabolomic profile before infestation differed from that of non-infested NM plants. This indicates that enhanced resistance may be supported by both an enhanced accumulation of defensive compounds before infection and a faster response after infestation by accumulating secondary metabolites that contribute to reduced mite performance. Among the compounds that accumulated over the priming profile in infested AM plants, we identified three flavonoids and their precursors diconyferil alcohol and cinnamic acid. Of note, flavonoids are involved in the defense against insects and pathogens (Lattanzio et al., 2006). Interestingly, the flavonoids naringenin and hesperetin and the alkaloid macarpine are also over-accumulated in *C. aurantium* plants infested with *T. urticae* (Agut et al., 2014). Unexpectedly, none of these flavonoid derivatives was over-accumulated in AM citrus.

It is known that the diverse family of flavonoids, among others, plays a key role in the defense against abiotic and biotic stressors (Falcone Ferreyra et al., 2012). Many of these compounds are direct scavengers of reactive oxygen species (Agati et al., 2012; Mierziak et al., 2014) and contribute to the alleviation of cellular damage caused by pathogen or insect attacks. Agut et al. (2014) reported that naringenin, hesperetin, and p-coumaric acid contribute to citrus resistance against *T. urticae*. Supporting these findings, Hoseinzadeh et al. (2020) found a high representation of flavonoid biosynthetic genes, flavonoid metabolic processes, and anthocyanin biosynthesis in an enrichment analysis of a comparative transcriptome between *Phaseolus* cultivars that were resistant or susceptible to *T. urticae*. To the best of our knowledge, no specific functions of the flavone luteolin, the flavanone neohesperidin, and the flavonol kaempferol have been previously related to mite resistance, although the flavonoid compounds are gaining relevance as essential players against the two-spotted spider mites. Noteworthy, these three flavonoids were over-accumulated in the locally infested leaves of mycorrhizal citrus. Strengthening this hypothesis, two phenols, diconiferyl alcohol and cinnamic acid, were accumulated in *C. aurantium* following a priming profile. These two compounds may be directly converted into phenylpropanoid compounds, flavonoids, or lignans (Le Roy et al., 2016). A functional characterization of the role of these compounds in MIR revealed that a mixture containing all of them at 0.5 mM was strongly effective, reducing leaf damage caused by herbivory. This, however, does not contradict the additional contribution of other metabolites that were not characterized in this study. In fact, the lignans pathway is also represented in the signals accumulated in AM vs. NM citrus in both local and systemic leaves. Lignans and, more specifically, yatein, have been recently proposed as markers for MIR against pathogens in tomato plants (Sanmartín et al., 2020). Of note, pharmacological approaches using a complex mixture of all identified compounds with a priming profile provided stronger protection of treated plants compared to mock-treated controls. This observation strengthens the hypothesis that MIR is mediated by the priming of specific secondary metabolites, providing an effective chemical defense based on a mixture of metabolites rather than on a single compound. This suggests that MIR may contribute to the control of spider mites infestation, compared with single-compound pesticides that are rapidly detoxified by mites.

In addition to phenolic derivatives, malic acid was also identified among the priming compounds in AM *C. aurantium*. This compound was previously identified in clusters of primed compounds in *Arabidopsis* plants treated with beta-aminobutyric acid (Pastor et al., 2014). Although its role in MIR in citrus remains unclear, it is likely that it mediates enhanced immune responses against the mite. The increase in oxocarboxylic acids, carbon metabolites, and amino acids in both AM and AMinf (24 hpi) plants may suggest a better nutritional status of the host, which contributes to better performance of the mite, as has been shown in other mycorrhized plants (Hoffmann and Schausberger, 2012; Patiño-Ruiz and Schausberger, 2014). However, this was not observed in AM *C. aurantium*; on the contrary, the pathway ontology classification showed an increased number of hits regarding phenylpropanoids and vitamins, suggesting a metabolic flux from carbon and amino acid precursors into more specialized secondary metabolites. This was clearly observed with vitamins that are highly represented at 24 and 48 hpi in AM plants. Among vitamins, riboflavin and B6 vitamers were also linked to MIR in previous studies (Rivero et al., 2015). Interestingly, the increase in vitamins seemed to be specific in locally infested leaves, whereas it was not observed in systemic leaves in which MIR is not functional.

Terpenoid compounds are among the most abundant metabolites in AM plants at 24 hpi and also fatty acid derivatives occur in systemic and locally infested leaves. These pathways comprise the most relevant volatile compounds or their precursors released after herbivory (Sharma et al., 2017). Although it is unlikely to find green leaf volatiles in the soluble fraction, the overrepresentation of fatty acids was indicative of the abundance of green leaf volatile precursors in AM plants. Surprisingly, mites in Y-tube choice assays did not show a preference of AM or NM plants, both in the presence or absence of conspecifics. This may be due to the strong antixenotic capacity of *C. aurantium* upon herbivory (Agut et al., 2015; Cabedo-López et al., 2019). Previous studies suggested that most changes in mycorrhized tomato plants have an impact on natural enemies rather than on pests (Hoffmann and Schausberger, 2012). Whether mycorrhizal citrus are more attractive to phytoseiids (the key *T. urticae* natural enemies) needs further studies. Unexpectedly, systemic treatments did not result in reduced mite performance in pre-infested mycorrhized plants. Despite the changes observed in the comparative metabolomic analysis between AM and NM pathways in systemic leaves, from our results, we conclude that in AM *C. aurantium*, either systemic soluble signals or volatile organic compounds do not increase deterrence against mite performance. This does not rule out the effectiveness of anticipating systemic responses in more susceptible citrus genotypes colonized by AMF.

Phytohormone regulation seems to be relevant in mycorrhized citrus defense against spider mites. As expected, expression of SA and JA-dependent marker genes was induced in naive citrus at early time points. Despite the induction of both pathways, Agut et al. (2014) demonstrated that resistance against *T. urticae* is mediated by oxylipins, whereas SA signaling seems to be dispensable. AM *C. aurantium* showed increased levels of JA, OPDA, and JA-Ile in the absence of infestation, whereas only JA and JA-Ile remained elevated after infection. This suggests a transformation of the precursor oxylipin OPDA into the more active forms of the hormones JA and JA-Ile during infestation. Accordingly, *LOX2* and *PR3* implicated in the oxylipin pathway, display a priming profile in AM citrus plants at 24 hpi. NM plants significantly increased the level of JA-dependent genes at 48 hpi as part of their innate immune response, as *C. aurantium* shows comparably strong basal resistance to the mite. This clearly indicates that despite innate resistance, mycorrhizal symbiosis triggers earlier responses in citrus, boosting the local defensive responses that are likely regulated by oxylipins, as has been shown in other mycorrhized plant species (Mora-Romero et al., 2015; Rivero et al., 2021). Flavonoids and secondary metabolites have a strong impact on defense against pathogens because of their antimicrobial activity, but they also reduce plant quality to insects, thus reducing the range of hosts (Burghardt et al., 2001). Previous studies confirmed that oxylipins and JA-related responses are tightly regulated in mycorrhized plants (Sanchez-Bel et al., 2018; Jiang et al., 2021; Rivero et al., 2021). *LOX* and *AOS* upregulation in myco rrhized poplar is correlated with an increase in 4-coumarate-CoA ligase, chalcone isomerase, and other flavonoid-related genes. In a previous study, we observed a positive correlation between oxylipins and flavonoids that supported enhanced resistance of *C. aurantium*, compared to other susceptible genotypes (Agut et al., 2014). In the present study, it is likely that priming of JA-dependent defenses was responsible for the observed increase in luteolin, neoshesperedin, kaempferol, coumaric acid, and diconiferyl alcohol upon mite herbivory; however, this hypothesis requires further research.

In conclusion, AM *C. aurantium* displayed enhanced resistance through antibiosis in local leaves, whereas its innate strong systemic resistance (Agut et al., 2016) was not boosted in systemic leaves. Although sugar and amino acid content increased in AM plants, which may increase host quality for arthropods, under our experimental conditions, some of these carbon sources from AM citrus seem to be converted into secondary metabolites that may sustain improved resistance against *T. urticae* in local leaves. In fact, exogenous treatments with priming compounds found in AM plants strongly reduced leaf damage rates. These rapid defense responses are likely coordinated by enhanced activity of JA-dependent defenses. Mycorrhization in *C. aurantium* led to reduced leaf damage and mite oviposition at the local level, whereas systemic responses and host selection by the pest remained unaltered, compared to NM plants. After determining the functionality of MIR in citrus defenses against spider mites, it is of considerable interest to extend such research to more susceptible genotypes which so far remain unexplored.

## Data Availability Statement

The datasets presented in this study can be found in online repositories. The names of the repository/repositories and accession number(s) can be found in the article/Supplementary Material.

## Author Contributions

MM-G performed the bioassays of local and systemic resistance, as well as part of the formal analysis, olfactometer assays, time course of the experiments and antibiotic assays, and maintenance of the spider mite and statistical analysis. JP-F performed the gene expression analysis and collaborated in the performance of the experiments. PS-B participated in the phenotypic assays, olfactometer assays, and hormone analysis. JJ contributed to the two choice assays and discussion and supervision of the manuscript. VP contributed the conceptualization, writing of the manuscript, supervision of the research, interpretation of hormonal assays, and funding acquisition. VF contributed with conceptualization, the writing of the manuscript, metabolomic analysis, and funding acquisition. All authors contributed to the article and approved the submitted version.

## Conflict of Interest

The authors declare that the research was conducted in the absence of any commercial or financial relationships that could be construed as a potential conflict of interest.

## Publisher’s Note

All claims expressed in this article are solely those of the authors and do not necessarily represent those of their affiliated organizations, or those of the publisher, the editors and the reviewers. Any product that may be evaluated in this article, or claim that may be made by its manufacturer, is not guaranteed or endorsed by the publisher.

## Footnotes

1
https://metlin.scripps.edu

2
https://pubchem.ncbi.nlm.nih.gov

3
https://massbank.eu

4
www.bioconductor.org

## Supplementary Material

Supplementary Figure 1 Root of mycorrhizal C. aurantium plant colonized by *Rhizophagus irregularis* fungi. *C. aurantium* plants were inoculated with 15% of the *R. irregularis* inoculum. 30 days after inoculation, random samples of mycorrhizal citrus roots were collected, stained and the percentage of total root colonization was measured by counting the number of **(A)** vesicles and **(B)** arbuscles.

Supplementary Figure 2 Pathways with clusters displaying a priming profile in locally infested leaves of C. *aurantium* plants. Evolution of clustering at 48 h post infestation among the pathways displaying a priming profile: Oxocarboxylic acids, (a primary metabolic pathway) and Flavonoids and flavonol biosynthesis (a secondary metabolic pathway). NM: non-mycorrhizal non-infested plants. AM: mycorrhizal non-infested plants. NM inf: non-mycorrhizal infested plants. AM inf: mycorrhizal infested plants. Signals of different treatments were compared after a Kruskal–Wallis test (*p* < 0.05) following adduct and isotope correction. Three independent and two technical replicates were randomly performed (*n* = 5).

Supplementary Figure 3 Effects of mycorrhization on the metabolic profile of systemic leaves of C. *aurantium.* Non-targeted principal component analysis (PCA) obtained from a non-target analysis by UPLC-QTOFMS of systemic leaves at **(A)** 24 h post infestation and **(B)** 48 h post infestation with *T. urticae. 3-*months-old plants were infested with 5 2-days-old female *T. urticae* (2 leaves per plant). 24 or 48 h after infestation, distal non-infested leaves were detached and frozen at –80°C. A pool from 6 individual plants was employed for each combination. Three independent and two technical replicates were randomly performed. Four different treatments were analyzed: NM: non-mycorrhizal non-infested plants. AM: mycorrhizal non-infested plants. NM inf: non-mycorrhizal infested plants. AM inf: mycorrhizal infested plants.

Supplementary Figure 4 Pathways with strong changes in systemic leaves of C. *aurantium*. Sector graph of pathways with signals more accumulated in AMinf/AM distal non-infested leaves compared with NMinf/NM at **(A)** 24 h post infestation and **(B)** 48 h post infestation. Four different treatments were employed: NM: non-mycorrhizal non-infested plants. AM: mycorrhizal non-infested plants. NM inf: non-mycorrhizal locally infested plants. AM inf: mycorrhizal locally infested plants. Pathways with more changes were obtained by the software MarVis (Marvis Pathway package). Signals from different treatments were compared after a Kruskal–Wallis test (*p* < 0.05) following adduct and isotope correction. A pool from 6 individual plants was employed for each combination. Three independent and two technical replicates were randomly performed (*n* = 5).

Supplementary Figure 5 Systemic responses of *C. aurantium* mycorrhizal plants against *T. urticae* after distal treatment. Relative damaged area on leaf surface. Half of the mycorrhizal and non-mycorrhizal plants were previously locally infested with 10 adult females per each plant. 72 h after the first treatment, all the plants were infested with 5 mites per systemic leaf (2 leaves per plant). 72 h after the second infestation, the damage rate was determined on the systemic leaves. Three treatments: AM (mycorrhizal plants with only the second infestation); NM inf (non-mycorrhizal plants with local and systemic infestation); AM inf (non-mycorrhizal plants with local and systemic infestation, were compared to the control: NM (Non-mycorrhizal plants with only the second infestation). Different letters indicate significant differences between treatments. The assay was repeated a minimum of three times. Asterisks indicate significant differences at *P* < 0.05 one asterisk, *P* < 0.01 two asterisks, *P* < 0.001 three asterisks for generalized linear model (GLIM, n = 6).
