# Supplementary figures and images for: Mycorrhizal Symbiosis Triggers Local Resistance in Citrus Plants Against Spider Mites
Source: Front Plant Sci. 2022 Jul 1;13:867778. doi: 10.3389/fpls.2022.867778 (PMC9285983; doi:10.3389/fpls.2022.867778)

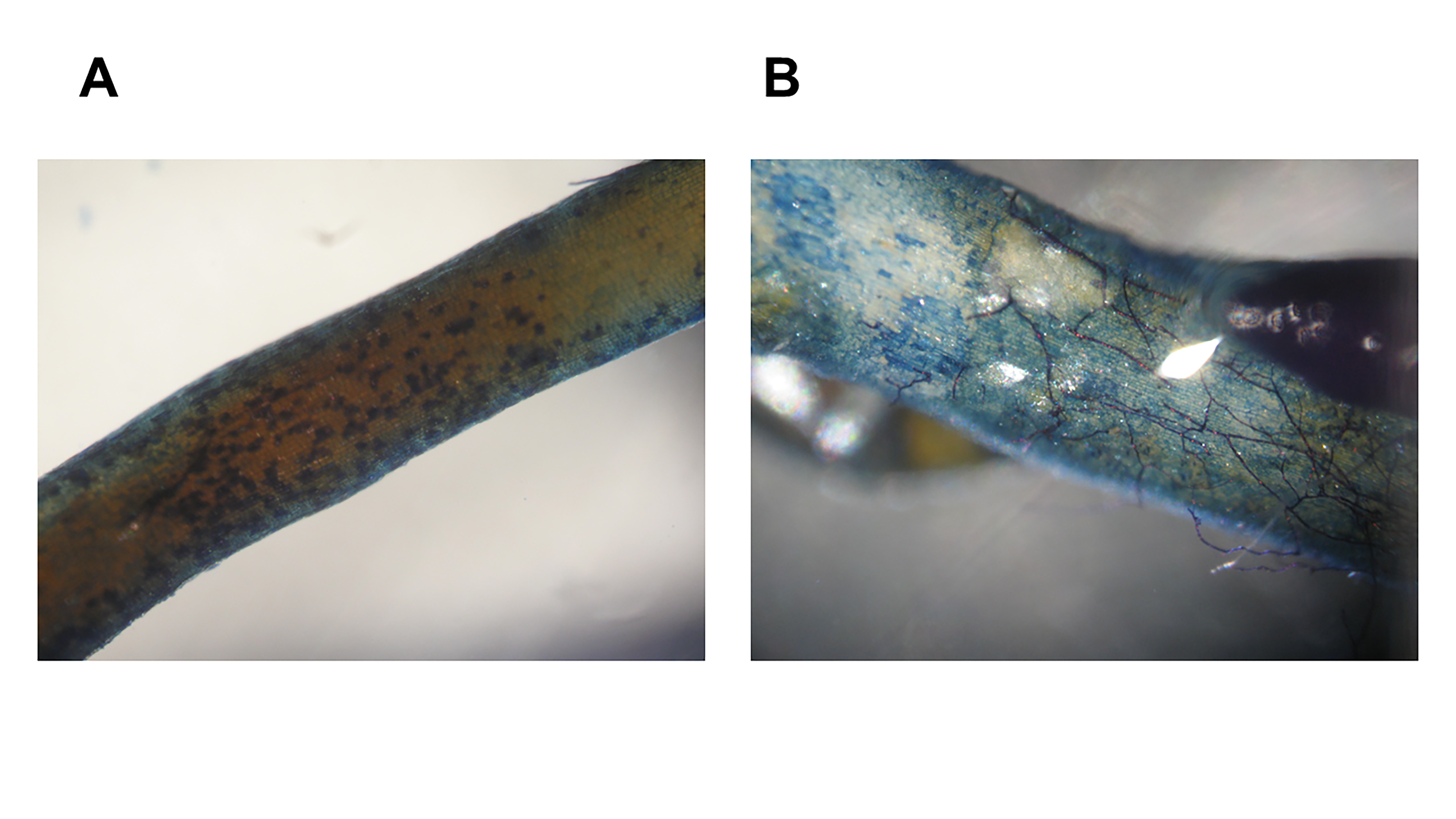

Supplement: Supplementary Figure 1 — Root of mycorrhizal C. aurantium plant colonized by Rhizophagus irregularis fungi. C. aurantium plants were inoculated with 15% of the R. irregularis inoculum. 30 days after inoculation, random samples of mycorrhizal citrus roots were collected, stained and the percentage of total root colonization was measured by counting the number of (A) vesicles and (B) arbuscles. [file Image_1.TIF]

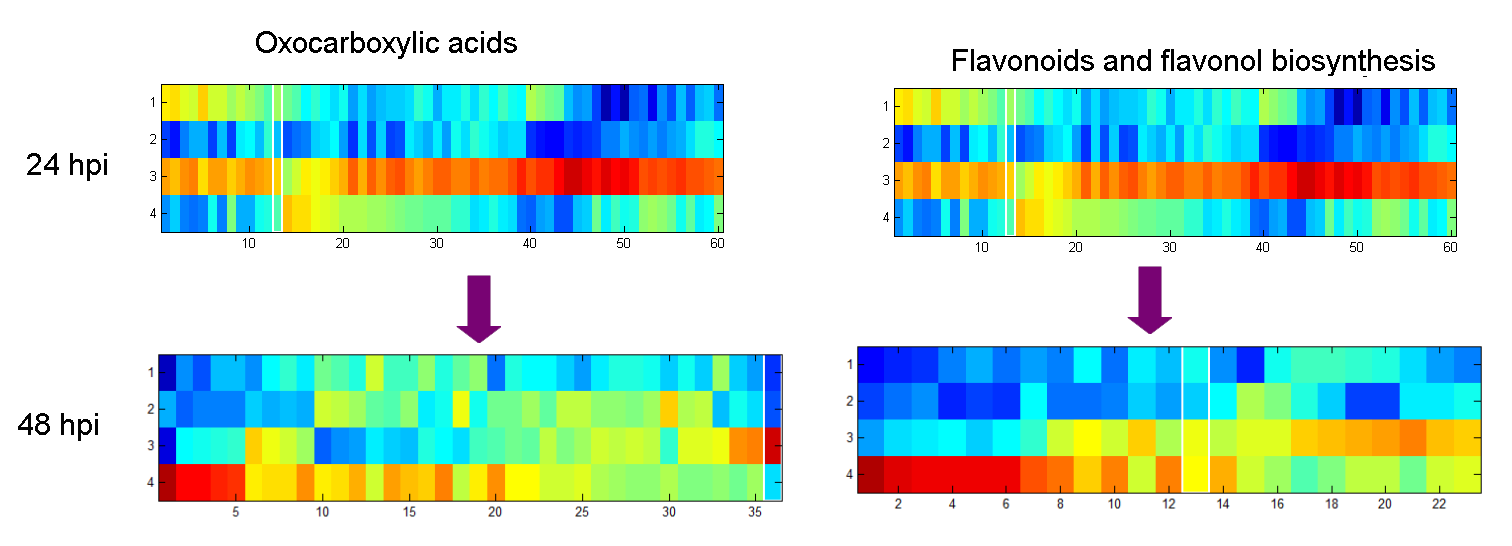

Supplement: Supplementary Figure 2 — Pathways with clusters displaying a priming profile in locally infested leaves of C. aurantium plants. Evolution of clustering at 48 h post infestation among the pathways displaying a priming profile: Oxocarboxylic acids, (a primary metabolic pathway) and Flavonoids and flavonol biosynthesis (a secondary metabolic pathway). NM: non-mycorrhizal non-infested plants. AM: mycorrhizal non-infested plants. NM inf: non-mycorrhizal infested plants. AM inf: mycorrhizal infested plants. Signals of different treatments were compared after a Kruskal–Wallis test (p < 0.05) following adduct and isotope correction. Three independent and two technical replicates were randomly performed (n = 5). [file Image_2.TIF]

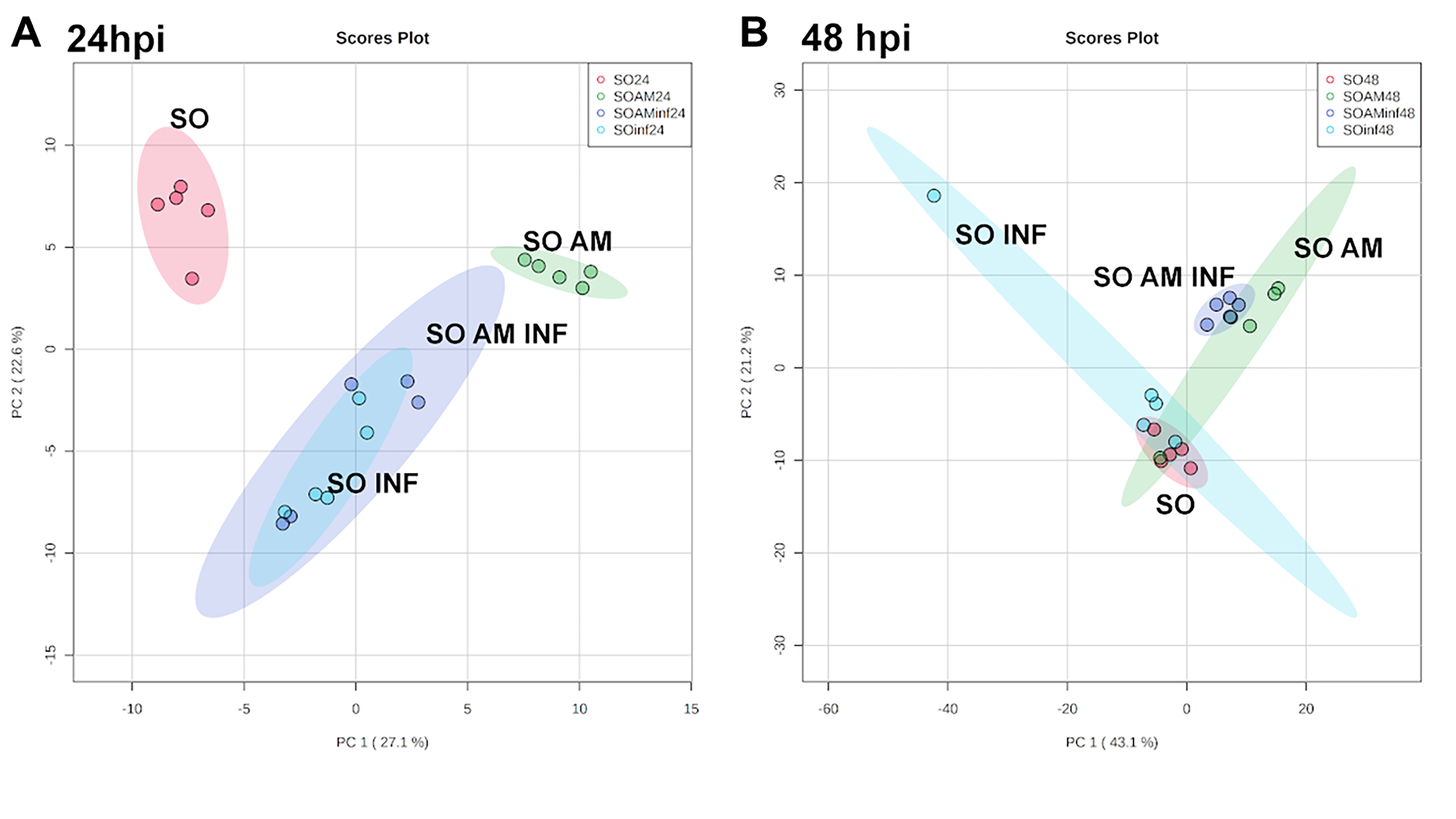

Supplement: Supplementary Figure 3 — Effects of mycorrhization on the metabolic profile of systemic leaves of C. aurantium. Non-targeted principal component analysis (PCA) obtained from a non-target analysis by UPLC-QTOFMS of systemic leaves at (A) 24 h post infestation and (B) 48 h post infestation with T. urticae. 3-months-old plants were infested with 5 2-days-old female T. urticae (2 leaves per plant). 24 or 48 h after infestation, distal non-infested leaves were detached and frozen at –80°C. A pool from 6 individual plants was employed for each combination. Three independent and two technical replicates were randomly performed. Four different treatments were analyzed: NM: non-mycorrhizal non-infested plants. AM: mycorrhizal non-infested plants. NM inf: non-mycorrhizal infested plants. AM inf: mycorrhizal infested plants. [file Image_3.TIF]

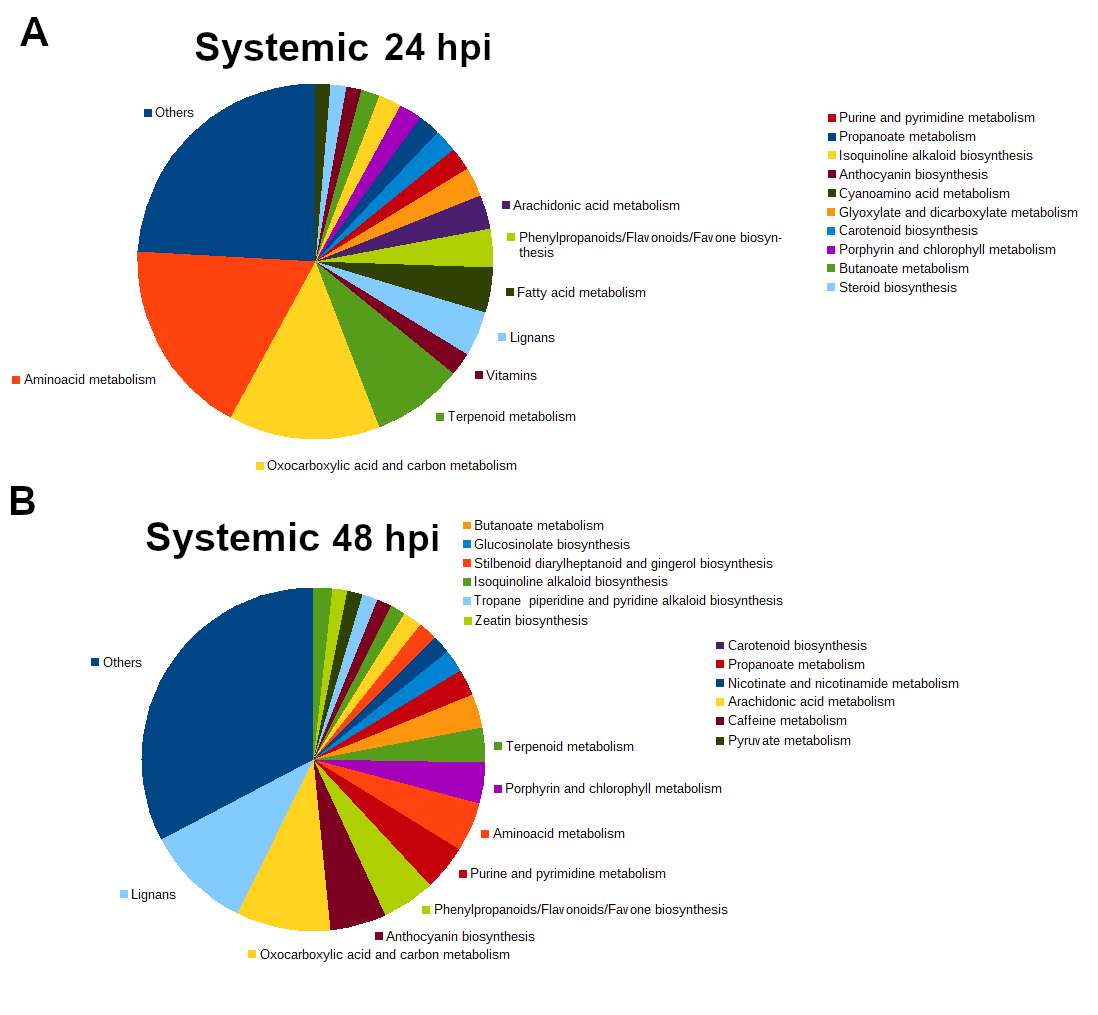

Supplement: Supplementary Figure 4 — Pathways with strong changes in systemic leaves of C. aurantium. Sector graph of pathways with signals more accumulated in AMinf/AM distal non-infested leaves compared with NMinf/NM at (A) 24 h post infestation and (B) 48 h post infestation. Four different treatments were employed: NM: non-mycorrhizal non-infested plants. AM: mycorrhizal non-infested plants. NM inf: non-mycorrhizal locally infested plants. AM inf: mycorrhizal locally infested plants. Pathways with more changes were obtained by the software MarVis (Marvis Pathway package). Signals from different treatments were compared after a Kruskal–Wallis test (p < 0.05) following adduct and isotope correction. A pool from 6 individual plants was employed for each combination. Three independent and two technical replicates were randomly performed (n = 5). [file Image_4.TIF]

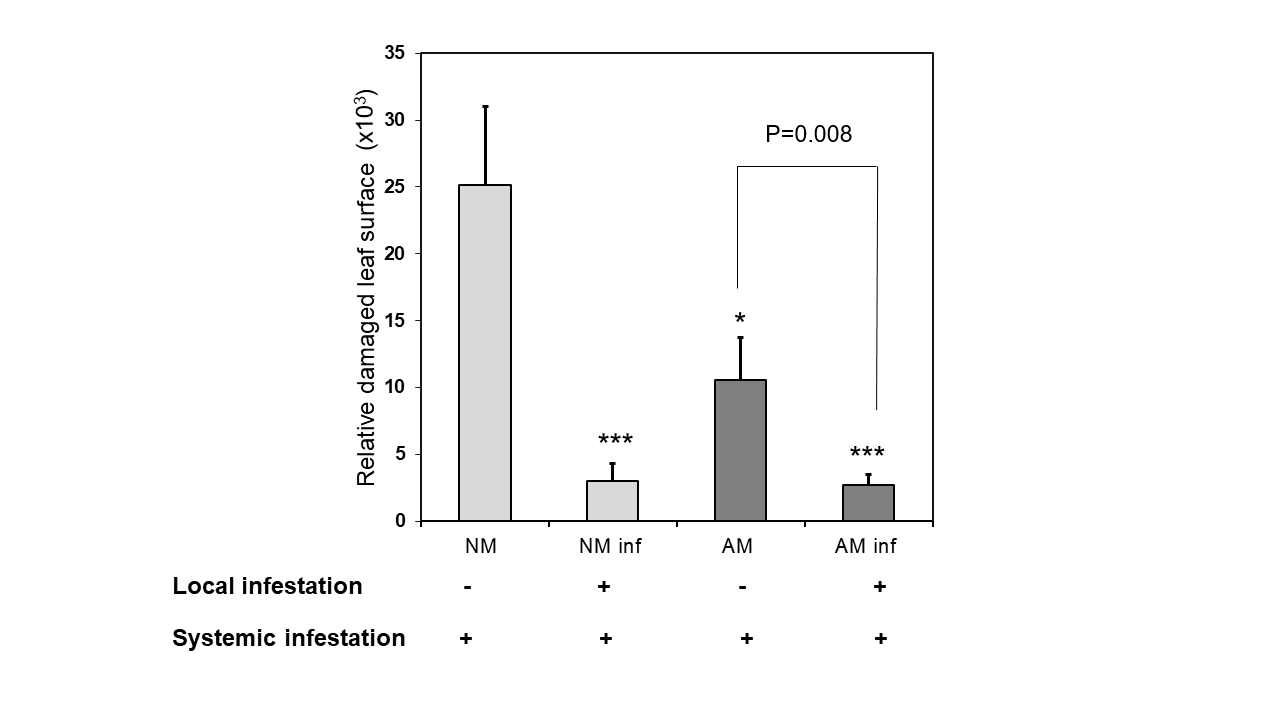

Supplement: Supplementary Figure 5 — Systemic responses of C. aurantium mycorrhizal plants against T. urticae after distal treatment. Relative damaged area on leaf surface. Half of the mycorrhizal and non-mycorrhizal plants were previously locally infested with 10 adult females per each plant. 72 h after the first treatment, all the plants were infested with 5 mites per systemic leaf (2 leaves per plant). 72 h after the second infestation, the damage rate was determined on the systemic leaves. Three treatments: AM (mycorrhizal plants with only the second infestation); NM inf (non-mycorrhizal plants with local and systemic infestation); AM inf (non-mycorrhizal plants with local and systemic infestation, were compared to the control: NM (Non-mycorrhizal plants with only the second infestation). Different letters indicate significant differences between treatments. The assay was repeated a minimum of three times. Asterisks indicate significant differences at P < 0.05 one asterisk, P < 0.01 two asterisks, P < 0.001 three asterisks for generalized linear model (GLIM, n = 6). [file Image_5.tif]
